# Regular Moderate or Intense Exercise Prevents Depression-Like Behavior without Change of Hippocampal Tryptophan Content in Chronically Tryptophan-Deficient and Stressed Mice

**DOI:** 10.1371/journal.pone.0066996

**Published:** 2013-07-04

**Authors:** Hosung Lee, Makoto Ohno, Shigeo Ohta, Toshio Mikami

**Affiliations:** 1 Department of Biochemistry and Cell Biology, Institute of Gerontology, Nippon Medical School, Nakahara-ku, Kawasaki, Japan; 2 Graduate School of Health and Sport Science, Nippon Sport Science University, Setagaya-ku, Tokyo, Japan; 3 Department of Health and Sports Science, Nippon Medical School, Nakahara-ku, Kawasaki, Kanagawa, Japan; VIB & Katholieke Universiteit Leuven, Belgium

## Abstract

Regular exercise has an antidepressant effect in human subjects. Studies using animals have suggested that the antidepressant effect of exercise is attributable to an increase of brain 5-hydroxytryptamine (5-HT); however, the precise mechanism underlying the antidepressant action via exercise is unclear. In contrast, the effect of 5-HT on antidepressant activity has not been clarified, in part because the therapeutic response to antidepressant drugs has a time lag in spite of the rapid increase of brain 5-HT upon administration of these drugs. This study was designed to investigate the contribution of brain 5-HT to the antidepressant effect of exercise. Mice were fed a tryptophan-deficient diet and stressed using chronic unpredictable stress (CUS) for 4 weeks with or without the performance of either moderate or intense exercise on a treadmill 3 days per week. The findings demonstrated that the onset of depression-like behavior is attributable not to chronic reduction of 5-HT but to chronic stress. Regular exercise, whether moderate or intense, prevents depression-like behavior with an improvement of adult hippocampal cell proliferation and survival and without the recovery of 5-HT. Concomitantly, the mice that exercised showed increased hippocampal noradrenaline. Regular exercise prevents the impairment of not long-term memory but short-term memory in a 5-HT-reduced state. Together, these findings suggest that: (1) chronic reduction of brain 5-HT may not contribute to the onset of depression-like behavior; (2) regular exercise, whether moderate or intense, prevents the onset of chronic stress-induced depression-like behavior independent of brain 5-HT and dependent on brain adrenaline; and (3) regular exercise prevents chronic tryptophan reduction-induced impairment of not long-term but short-term memory.

## Introduction

Major depression is attributable to neurobiological and environmental factors [1]. The depletion of serotonin (5-hydroxytryptamine; 5-HT) is regarded as the most potent neurobiological factor in the etiology of depression [2]. Several disturbances of the 5-HT system have been reported in depression, including decreased plasma tryptophan [3] and decreased serotonin 5-HT, in postmortem brains of depressed patients [4]. As the availability of plasma tryptophan, the precursor of 5-HT, is a limiting factor in the synthesis of brain 5-HT [2], acute tryptophan depletion (ATD) is used to study the effects of 5-HT depletion on the onset of depression in humans and rodents. ATD results in a lowering of plasma and brain tryptophan [5] and a decrease of brain 5-HT synthesis [6] concomitantly with changes in cognitive functions and depression- and anxiety-like behavior in rats [7] and transient mood effects in humans [8]. However, the ATD-induced effects are transient, while the decrease of 5-HT and mood disorders observed in depressed patients are chronic; therefore, we considered that the contribution of 5-HT depletion to the etiology of depression should be examined using long-term depletion in brain 5-HT. No previous study has examined the effect of long-term depletion of brain tryptophan and 5-HT on the onset and development of depression.

Stress is one of the most potent environmental factors for the induction and development of depression [2]. Stress raises the activity of the hypothalamic-pituitary-adrenal (HPA) axis and increases corticotrophin-releasing factor (CRF) and corticosteroid [9]. Furthermore, the persistence of stress and corticosteroid induces neural atrophy in limbic structures, mainly the hippocampus, and reduces cell proliferation and neurogenesis in the hippocampus [10], [11]. These changes lead to the onset and development of depression [12]. In addition, chronic stress impairs cognitive function, with depressive patients showing cognitive disturbances such as impairments in attention, working memory and executive function [13].

Exercise training improves psychological risk factors, including depression, anxiety, hostility, and total psychological stress, as well as stress-related mortality [14], and has been shown to improve depressive symptoms when used as an adjunct to medication [15]. In rodents, regular exercise was also shown to improve depression-like behavior in chronically stressed mice [16]. Nevertheless, the mechanisms underlying the antidepressant effect of exercise are not understood. It is possible that exercise could contribute to rescue the lowered level of brain 5-HT in depressive patients; however, the relationship between exercise-induced improvement of depression and the level of brain 5-HT has yet to be elucidated.

This study was designed to answer the following questions: (1) Is chronic tryptophan deficiency related to the onset and development of depression concomitantly with a deficit of cognitive function? (2) Are exercise-induced antidepressant effects related to brain 5-HT metabolism? To resolve these issues, mice fed a TD diet were subjected to 4 weeks of chronic mild stress (CUS) with or without the performance of treadmill running at either moderate or high intensity. Concomitantly, the mice were subjected to behavioral tests to examine depression-like behavior and cognitive function. Our findings demonstrated that the onset of depression-like behavior is attributable not to TD but to chronic stress, whereas TD triggered the impairment of cognitive function, and that regular exercise, whether moderate or intense, prevents depression-like behavior concomitantly with an improvement of hippocampal neurogenesis and an increase of hippocampal noradrenaline, despite the recovery of brain 5-HT.

## Materials and Methods

### 1. Animals and Diet

All experimental procedures and animal treatments were performed in accordance with the guidelines of the laboratory animal manual of Nippon Medical School. Male C57BL/6J mice (Sankyo Lab Service, Tokyo, Japan), aged 7 weeks and weighing 22.1±1.3 g, were used for this study. The mice were randomly divided into five groups: control mice (*C* mice; n = 10), tryptophan-deficiency (TD) mice (*TD* mice; n = 10), TD+chronic unpredictable stress (CUS) mice (*TD+CUS* mice; n = 10), TD+CUS+moderate exercise (ME) mice (*TD+CUS+ME* mice; n = 10) and TD+CUS+intense exercise (IE) mice (*TD+CUS+IE* mice; n = 10). As our previous study [16] indicated that mice fed a normal diet with exposure to CUS showed depressive behavior, in this study, we omitted the experimental condition concerning the mice fed a normal diet with exposure to CUS. The *C* and *TD* mice were housed in standard mouse cages with five mice per cage. The *TD+CUS*, *TD+CUS+ME* and *TD+CUS+IE* mice were housed in standard mouse cages divided into six cells to reduce their living space and to decrease their daily activity [17]. The *TD*, *TD+CUS*, *TD+CUS+ME* and *TD+CUS+IE* mice were fed a tryptophan-deficient (TD) powered diet (Oriental Yeast Co., Ltd., Japan). The *C* mice were fed a TD powered diet supplemented with tryptophan at 214 mg per 100 g of powder diet, equal to the tryptophan content in a standard animal diet. All diets were mixed with hot water, kneaded, cut and dried to make hard pellets. Tap water was given to all mice. All mice were introduced to their respective diets for a week before the start of the CUS procedure. During the experimental period, all mice were allowed to eat and drink ad libitum. The weight of all mice was measured once a week throughout the experimental period.

### 2. Chronic Unpredictable Stress

**Figure 1:**
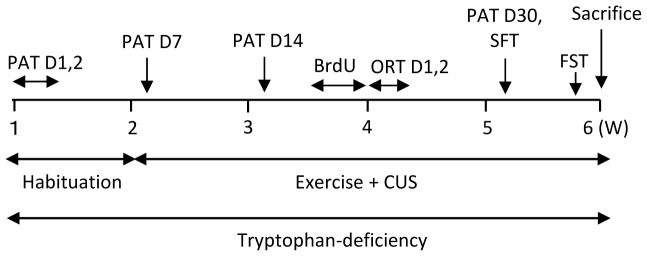
Experimental procedures. Habituation: habituation to cage, food and treadmill running; CUS: chronic unpredictable stress; PAT: passive avoidance test; ORT: object recognition test; SFT: sucrose preference test; FST: forced swimming test.

The timeline of the various experimental procedures is shown in Fig. 1. After 7 days of acclimation to cage and diet, all mice were subjected to a passive avoidance test (PAT) to examine learning and memory prior to CUS exposure. The protocol of PAT is described in the section on the behavior tests. After PAT, *TD+CUS*, *TD+CUS+ME* and *TD+CUS+IE* mice were exposed to 28 days of CUS according to a modified version of the method of Banasr et al. [18]. They were exposed to two stressors daily, as described in Table 1, in the morning and at night.

**Table 1:** Protocol of chronic unpredictable stress.

Day time stress	Times	Overnight stress	Times
Immobilization; 3 h	6	Light on overnigh	5
Cold isolation**(**°C**)**; 3 h	5	Web bedding overnigh	4
Cage rotation (100 rpm); 3 h	5	Crowding overnight	4
Swim in water**(**18°C**)**; 5 min	3	Food/water deprivation overnight	4
Rat odor; 3 h	4	Tilt of cage	5
Confrontation with rat; 3 h	5	Stroboscope overnight	5
Total	28	Total	27

### 3. Exercise

During the habituation period, all mice were subjected to 10 min of treadmill running at a treadmill speed of 10 m/min daily for five consecutive days to familiarize them with such running.

During the CUS procedure, *TD+CUS+ME* and *TD+CUS+IE* mice were subjected to moderate or intense running on a treadmill three days per week at 4∶00–6∶00 p.m. *TD+CUS+ME* mice were subjected to 1 h of running at a treadmill speed of 20 m/min. *TD+CUS+IE* mice were subjected to intense and intermittent exercise as follows: At first, mice performed running at a treadmill speed of 30 m/min for 1 min and then rested for 10 sec, which was defined as one set. After mice performed three sets of running at the same treadmill speed, the treadmill speed was increased to 35 m/min, and mice again performed three sets. In this way, the treadmill speed was increased at intervals of 5 m/min until the mice were exhausted.

### 4. 5-bromo-2-deoxyuridine (BrdU) Injection

Two weeks before the sacrifice of the animals, BrdU dissolved in sterilized saline was intraperitoneally injected into all mice at 50 mg/kg body weight for five consecutive days at 24-hour intervals in order to evaluate the survival of newly born cells.

### 5. Behavioral Tests

#### (1) Forced swimming test (FST)

All mice were subjected to FST to evaluate depression-like behavior according to the method of Porsolt et al. [19]. For testing, cylinders (height, 2 cm; diameter, 15 cm) filled with water (25°C) were used to make the mice swim or float without touching their hindlimbs or tail on the bottom of the cylinder. Each mouse was individually placed in the cylinder and its movements were recorded for 6 min using a video camera. Immobility time, when the mouse performed the minimal movement required to stay afloat, was measured to evaluate depression-like behavior during the latter four minutes of the test.

#### (2) Sucrose preference test (SFT)

At the end of 28 days of CUS, all mice were subjected to SFT to evaluate depression-like behavior according to the method of Sakata et al. [20]. In brief, after animals had been habituated to two water bottles for 3 days in their home cages, a free choice between plain water and 1% sucrose solution was provided to each mouse. The positions of the bottles were counterbalanced across the left and right sides of the testing cages. Water and sucrose intakes were measured during the 12-h dark period by weighing bottles before and after the test. Tests were performed on 2 consecutive days. Sucrose preference was calculated as the percentage of sucrose consumed.

#### (3) Passive avoidance test (PAT)

After 7 days of acclimation to cage and diet, all mice were subjected to PAT to examine the basal performance of learning and memory prior to CUS exposure. The apparatus for this test consisted of two compartments, one light and the other dark, separated by a vertical sliding door [21]. A mouse was initially placed in the light compartment for 30 sec. Then, the door was opened to permit the mouse to enter the dark compartment. After the mouse entered the dark compartment, the door was closed. Thirty seconds later, the mouse was given a 0.2 mA electric shock for 2 sec. The mouse was allowed to recover for 30 sec and then returned to the home cage. Twenty-four hours later, the mouse was again placed in the light compartment and the door was opened. The latency time until the mouse stepped through the door was determined as an index of learning and memory. To examine time-course changes in learning and memory, the latency to enter the dark compartment was measured every week during the CUS procedure.

#### (4) Object recognition test (ORT)

ORT was used to examine recognition memory [22], [23]. After the mice were transferred to a cage for the ORT and acclimated for 24 h, they were exposed to two differently shaped objects for 10 min. The number of actions of exploring and/or sniffing two objects was counted for the initial 5-min period (Training). The next day, to examine memory retention, one of the original objects was replaced with a novel one with a different shape, and then the number of actions of exploring and/or sniffing the novel object was counted for 5 min (Retention). The recognition index was calculated by dividing the number of actions of exploring and/or sniffing the novel object by the total number of actions of exploring and/or sniffing (novel object+familiar object) [23].

### 6. Immunohistochemistry

#### (1). Sample collection

The day after completion of all behavioral tests, the mice were anesthetized with pentobarbital and transcardially perfused with 60 ml of saline through the left ventricle. Brains were carefully removed and divided into their two hemispheres. The left hemisphere was fixed in 4% paraformaldehyde in 0.1 M phosphate-buffered saline (PBS; 137 mM NaCl, 8.10 mM Na_2_HPO_4_, 2.68 mM KCl, 1.47 mM KH_2_PO_4_, pH7.4) overnight at room temperature. After being washed three times with PBS, the brain was cut rostro-caudally with a Leica vibratome (VT 1000S, Leica Microsystems, Germany) at 40 µm. Serial sections were immersed in PBS. Ninety-six-well plates were used to maintain the correct order of the sections in PBS at 4°C. The right hemisphere was divided into the hippocampus, cerebral cortex, hypothalamus and cerebellum. These samples were quickly frozen in liquid nitrogen and stored at −80°C until analysis.

#### (2) BrdU and Ki67

BrdU- or Ki67-positive cells were identified immunohistochemically. The sections were incubated with 3% hydrogen peroxide in methanol to block endogenous peroxidase activity. BrdU sections were incubated with 2 M HCl for 30 min at 37°C and M.O.M. mouse IgG blocking solution for 1 h. Ki67 sections were exposed to heat (100°C) in 100 mM citric acid buffer (pH 6.0) for 5 min using a microwave for antigen retrieval and the sections were then blocked with normal goat serum. After washing with PBS, the sections were incubated for two nights with the primary antibody, a mouse monoclonal anti-BruU antibody (BD Pharmingen, 1∶200) or rabbit polyclonal anti-Ki67 antibody (Abcam, 1∶500). After washing, the BrdU or Ki67 sections were incubated with anti-mouse biotinylated IgG secondary antibody (Vector Laboratories, 1∶250) or goat anti-rabbit biotinylated IgG (Vector Laboratories, 1∶100) for 2 h at room temperature, respectively. Both BrdU and Ki67 sections were incubated with VECTASTAIN ABC reagent (Vector Laboratories) for 90 min and developed using 3,3′-diaminobenzidine (DAB).

#### (3) Cell quantification procedures

The sections reacted with antibody were mounted, dehydrated, and coverslipped using Permount mounting medium. We quantified the number of BrdU- or Ki67-positive cells according to Trejo et al. [24]. In brief, five sections were chosen from the region, which were located from 1.28 mm to 1.68 mm posterior to the bregma, and the density of BrdU- or Ki67-positive cells in the subgranular zone (SGZ), which is a region with a diameter 2–3 cells thick located between the granule cell layer and the hilus of the dentate gyrus, was calculated using a Leica DM3000 microscope (Leica, Germany) with a 40× objective. The same areas and number of sections were studied for all the animals and all the experimental groups. The areas of hippocampal dentate gyrus were also measured using NIH Image-J software and the cell density per mm^3^ was calculated.

### 8. Neurochemical Analysis

The levels of tryptophan, 5-HT and 5-hydroxyindole acetic acid (5-HIAA) in brain were analyzed according to a modified version of the method of Zhang et al. using high-performance liquid chromatography (HPLC). [25]. In brief, 100 mg of hippocampus tissue was homogenized in 0.5 ml of 0.2 M perchloric acid (PCA) containing 100 µM EDTA·2Na and 1 µg/ml DL-isoproterenol hydrochloride. The homogenate was centrifuged at 15,000 rpm for 15 min at 4°C. The supernatant was then neutralized to pH 3.0 by adding 1 M acetate and filtered with a 0.45 µm pore membrane filter, and then 20 µl of the filtrate was injected into a high-performance liquid chromatography (HPLC) system equipped with a EICOMPACK SC-50DS (φ 3.0 mm×150 mm) (EICOM, Tokyo) column. 0.1 M acetate/citrate containing 17% methanol, 190 µg/ml 1-octanesulfonic acid sodium salt, and 5 µg/EDTA·2Na (pH 3.0) was used as the mobile phase and kept at a constant flow of 0.5 ml/min. The column elute was monitored using an EPC-700 electrochemical detector (EICOM, Tokyo, Japan) and analyzed using PowerChrom EPC-500 software (EICOM, Tokyo, Japan).

### 9. Statistical Analysis

Data are presented as means ± S.E. Statistical analysis was performed using repeated measures ANOVA or factorial ANOVA as appropriate. To characterize differences between groups further, Tukey's post hoc test was used. A value of p<0.05 was accepted as the level of significance.

## Results

### 1. Body Weight

**Figure 2:**
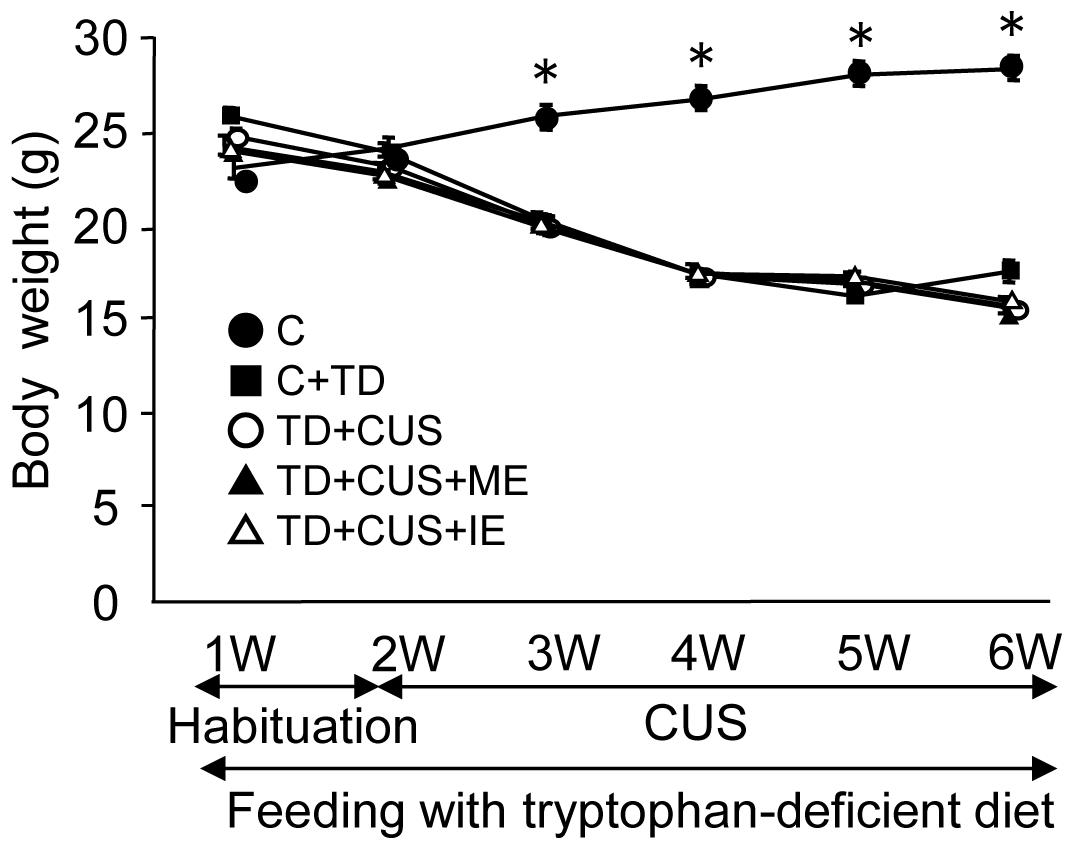
Effects of tryptophan deficiency, CUS and regular exercise on body weight. Data are expressed as mean ± SEM. *, p<0.05 vs. TD, TD+CUS, TD+CUS+ME, TD+CUS+IE; •, C; ▪, TD; **○**, TD+CUS; ▴, TD+CUS+ME; △, TD+CUS+ IE.

We measured the body weight before feeding on TD diet, at the start of CUS and at weekly intervals during the CUS procedure. The time-course change of body weight during the CUS protocol is shown in Fig. 2. At the beginning of the experiment, there was no difference in body weight among the groups of mice. During the experiment, the *C* mice gradually increased in body weight. In contrast, the mice fed a TD diet (*TD*, *TD+CUS*, *TD+CUS+ME* and *TD+CUS+IE* mice) gradually lost weight and showed a significantly lower body weight, except the control mice (p<0.05) (F [4, 47] = 186.749; P<0.001) (Fig. 2). However, there was no significant difference in body weight among the other groups of mice.

### 2. Neurochemical Results

**Figure 3:**
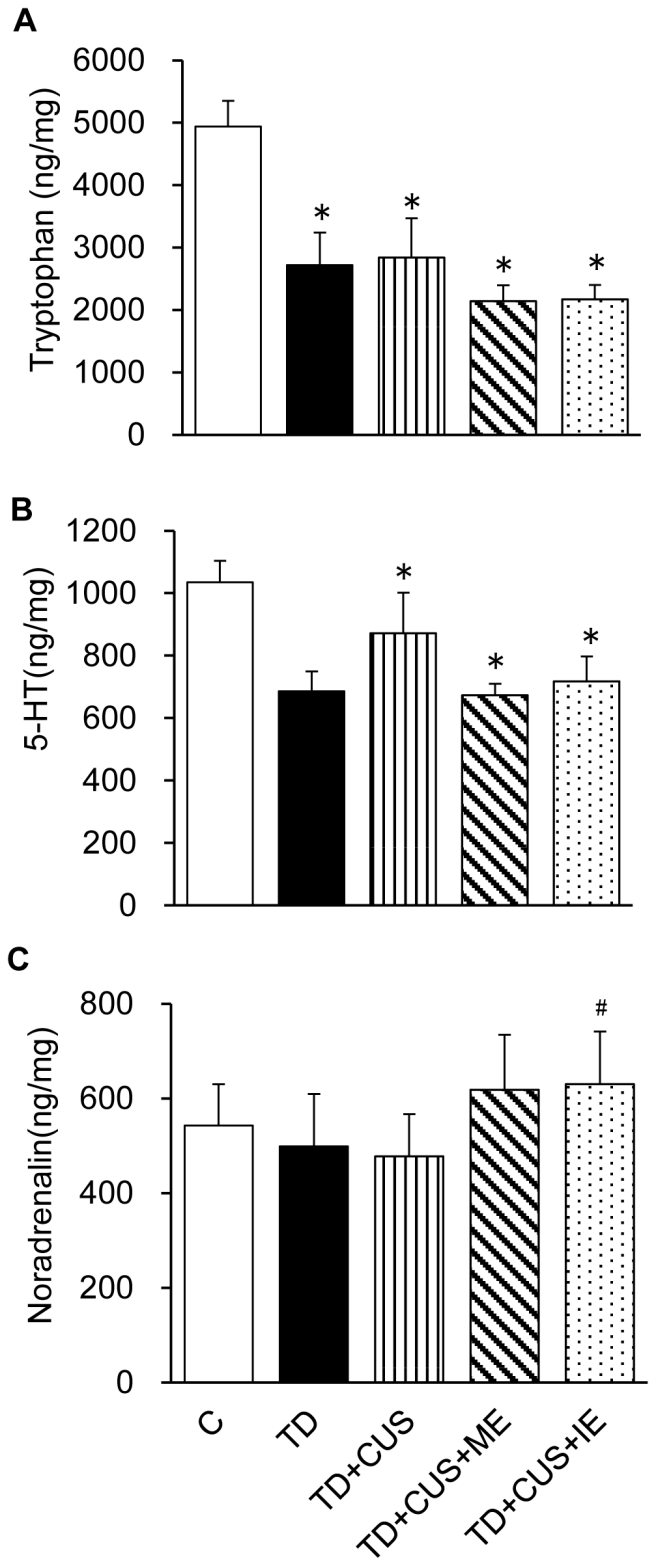
Effects of tryptophan deficiency, CUS and regular exercise on the levels of tryptophan (a), 5-HT (b) and noradrenaline (c) in the hippocampus. Data are expressed as mean ± SEM. *_,_ p<0.05 vs. C; #, p<0.05 vs. C, TD, TD+CUS.

The mice fed a TD diet showed significantly decreased hippocampal levels of tryptophan and 5–HT compared with the C mice (p<0.05) (Tryptophan: F [4], [42] = 6.813; P<0.000, 5-HT: F [4], [42] = 3.355; P<0.018) (Fig. 3a, b). Regular exercise, whether moderate or intense, did not restore the hippocampal levels of tryptophan and 5–HT to the control level. *TD+CUS+IE* mice showed significantly higher noradrenaline levels than the other groups of mice (F [4], [42] = 3.449; P<0.043) (Fig. 3c).

### 3. Behavioral Results

**Figure 4:**
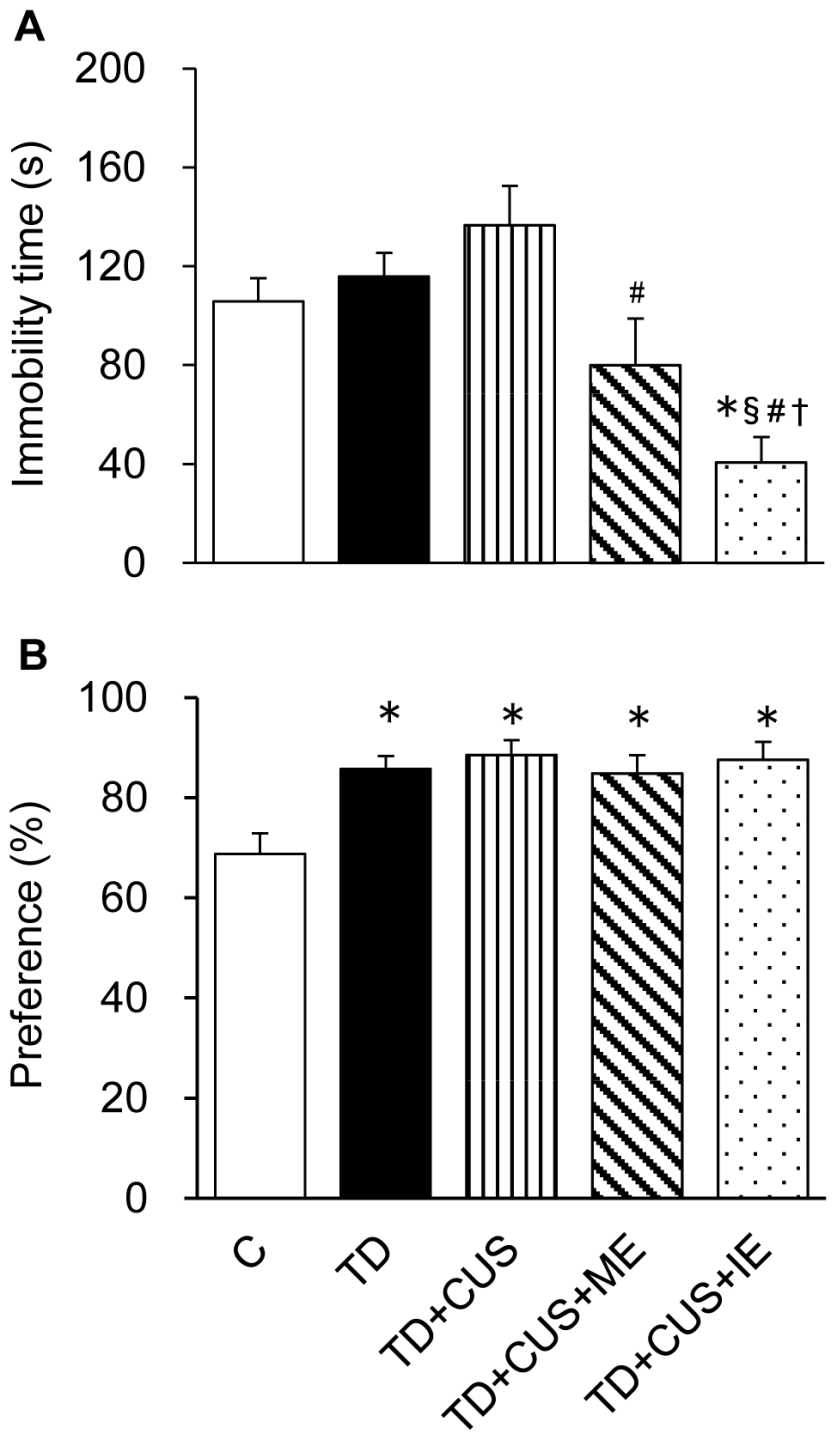
Effects of tryptophan deficiency, CUS and regular exercise in FST (a) and SFT (b). Data are expressed as mean ± SEM. *, p<0.05 vs. C; **§**, p<0.05 vs. TD; #, p<0.05 vs. TD+CUS; †, p<0.05 vs. TD+CUS+ME.

In FST (Fig. 4a), *TD+CUS* mice showed a higher immobility time than *C* and *TD* mice; however, there was no significant difference between *C* and *TD* mice. *TD+CUS+ME* and *TD+CUS+IE* mice showed significantly lower immobility time than *TD+CUS* mice. *TD+CUS+IE* mice showed significantly lower immobility time than the other groups (F [4], [43] = 6.75; P<0.001). These findings suggested that depression-like behavior is attributable not to TD but to CUS, and that regular exercise, whether moderate or intense, prevented depression-like behavior induced by CUS.

In SFT, the sucrose preference ratio of the control mice corresponded to the level of the sucrose preference ratio (70%) of the non-stressed mice fed a normal diet, as reported in a previous study [20]. The mice fed a TD diet showed significantly higher sucrose preference than the C mice (p<0.05) (F [4, 48] = 5.592; P<0.001) (Fig. 4b). These findings suggested that TD could enhance sucrose preference with or without chronic stress.

**Figure 5:**
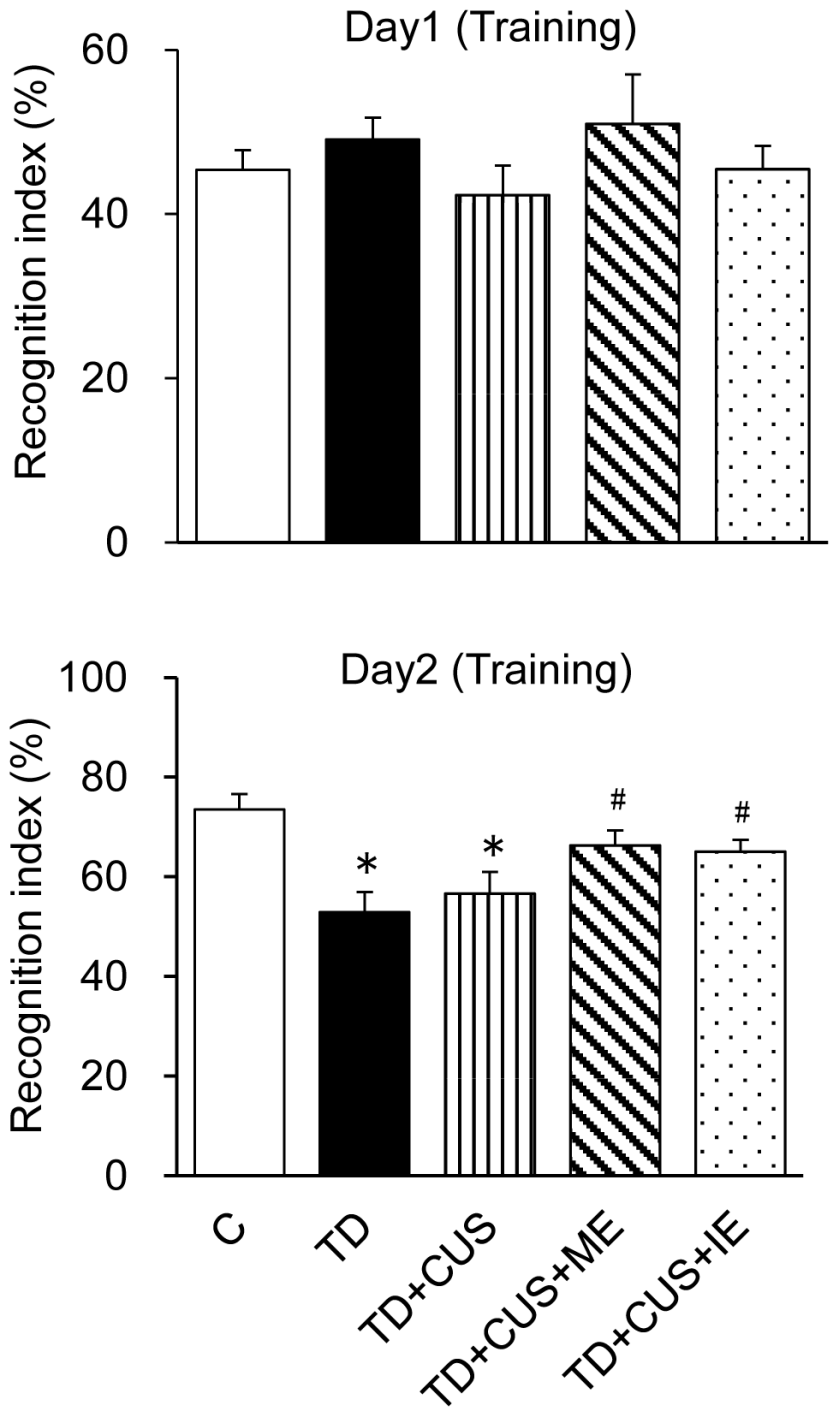
Effects of tryptophan deficiency, CUS and regular exercise on recognition index in object recognition. **Data are expressed as mean ± SEM.** *, p<0.05 vs. C; #, p<0.05 vs. TD.

To examine the effect of TD feeding and CUS on learning and memory, all mice were subjected to ORT in the 4th week of the experiment. *C* mice showed a strong preference for a novel object, whereas *TD* and *TD+CUS* mice showed a significantly decreased preference for a novel object compared with the *C* mice. There was no significant difference among *C*, *TD+CUS+ME* and *TD+CUS+IE* mice. In addition, *TD+CUS+ME* and *TD+CUS+IE* mice showed a significantly stronger preference for a novel object than *TD* mice (F [4], [45] = 5.701; P<0.001) (Fig. 5). These findings suggested that the impairment of cognitive ability was attributable not to CUS but to TD, and that regular exercise, whether moderate or intense, prevented the impairment of learning and memory.

**Figure 6:**
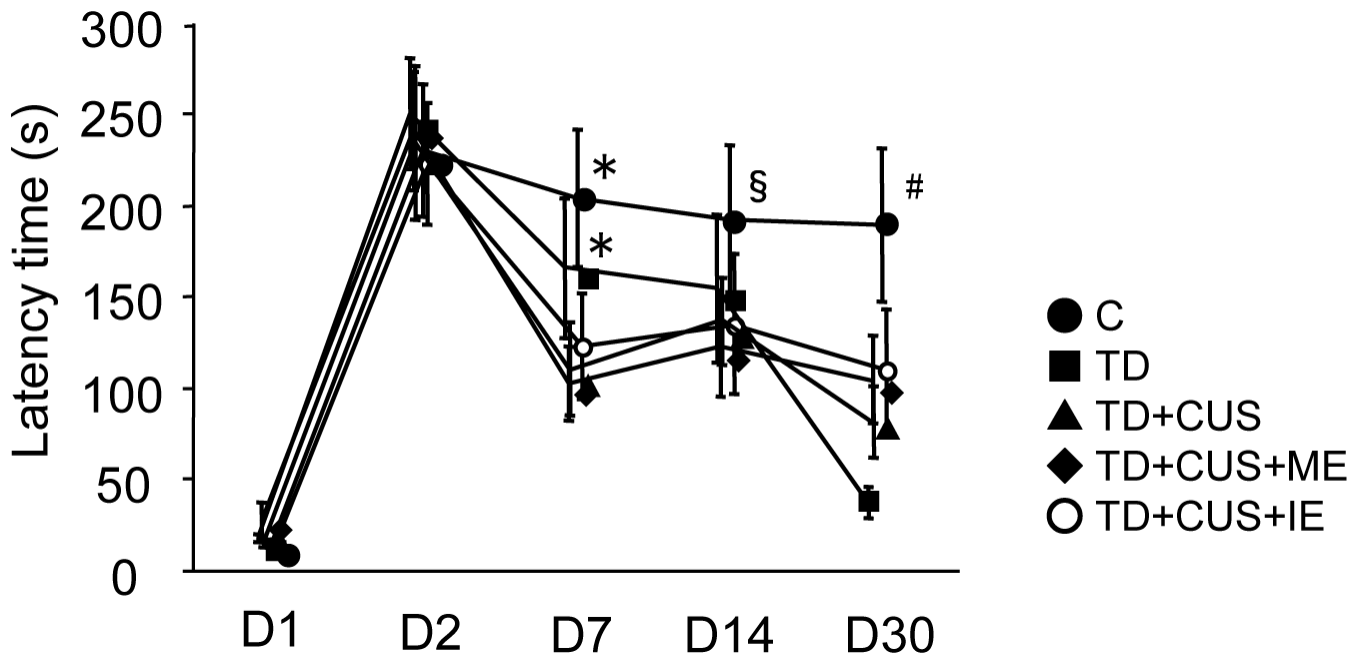
Effects of tryptophan deficiency, CUS and regular exercise on latency time to re-enter a dark compartment in the passive avoidance test. Data are expressed as mean ± SEM. *, p<0.05 vs. TD+CUS, TD+CUS+IE; §, p<0.05 vs. TD+CUS+IE; #, p<0.05 vs. TD, TD+CUS, TD+CUS+ME, TD+CUS+IE.

Moreover, we exposed the mice to PAT for examination of the effects of TD and CUS on long-term memory. When latency to reenter the dark compartment was measured before CUS treatment, all the groups of mice showed equal values (over 200 sec). Subsequently, *C* mice maintained constant latency to reenter the dark compartment when it was re-measured on the 7th, 14th and 30th days after the training day. In contrast, other groups of mice showed gradual decreases in latency and showed significantly decreased latency compared with *C* mice on the 30th day (F [4, 46] = 4.697; P<0.003) (Fig. 6). These findings suggested that the impairment of long-term memory is attributable not to CUS but to TD, and that regular exercise, whether moderate or intense, does not prevent the impairment of long-term memory.

### 3. Immunohistochemical Results

**Figure 7:**
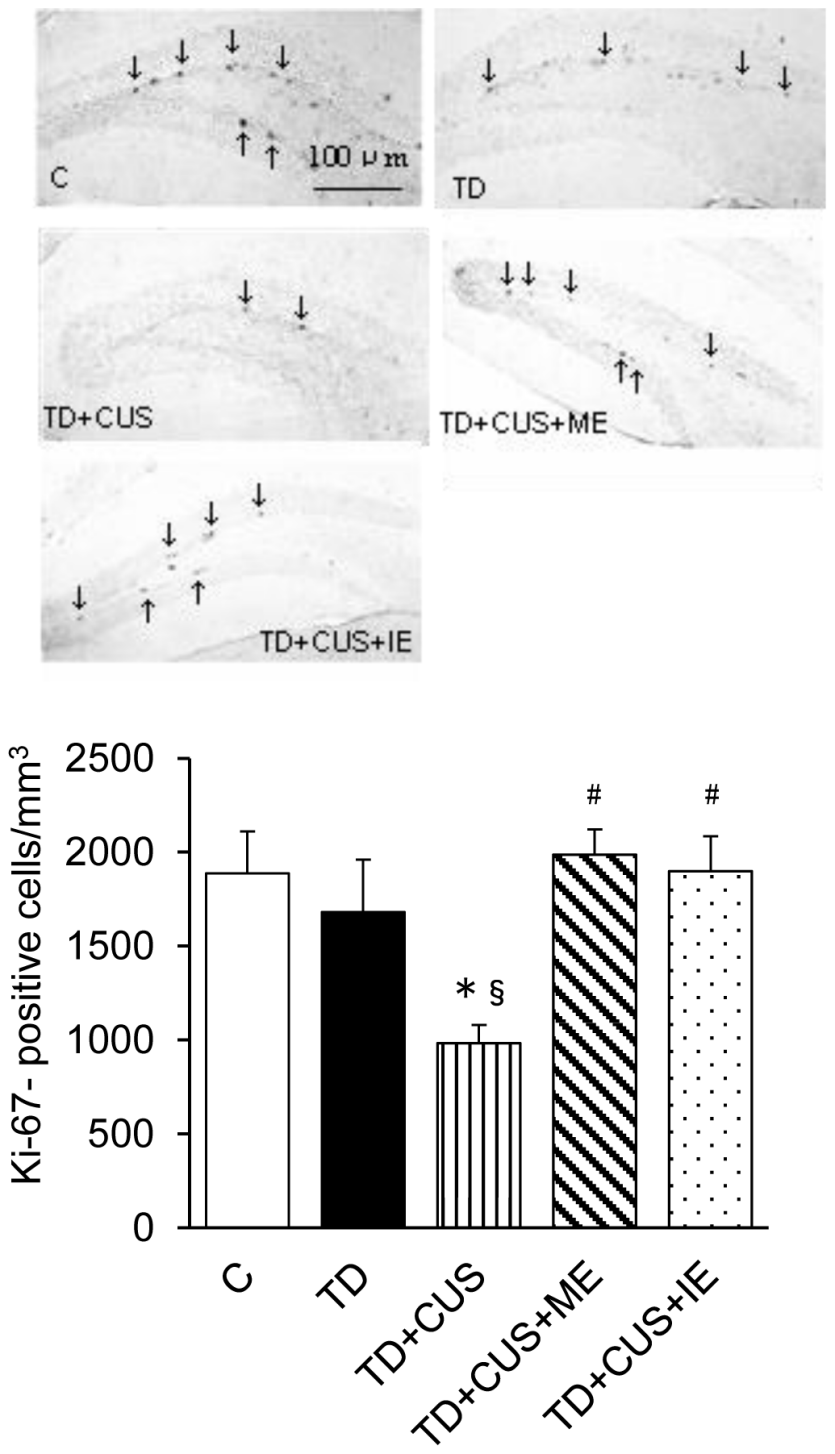
Effects of tryptophan deficiency, CUS and regular exercise on number of Ki67-positive cells in the dentate gyrus of the hippocampus. Data are expressed as mean ± SEM. *, p<0.05 vs. C; §, p<0.05 vs. TD; #, p<0.05 vs. TD+CUS.

**Figure 8:**
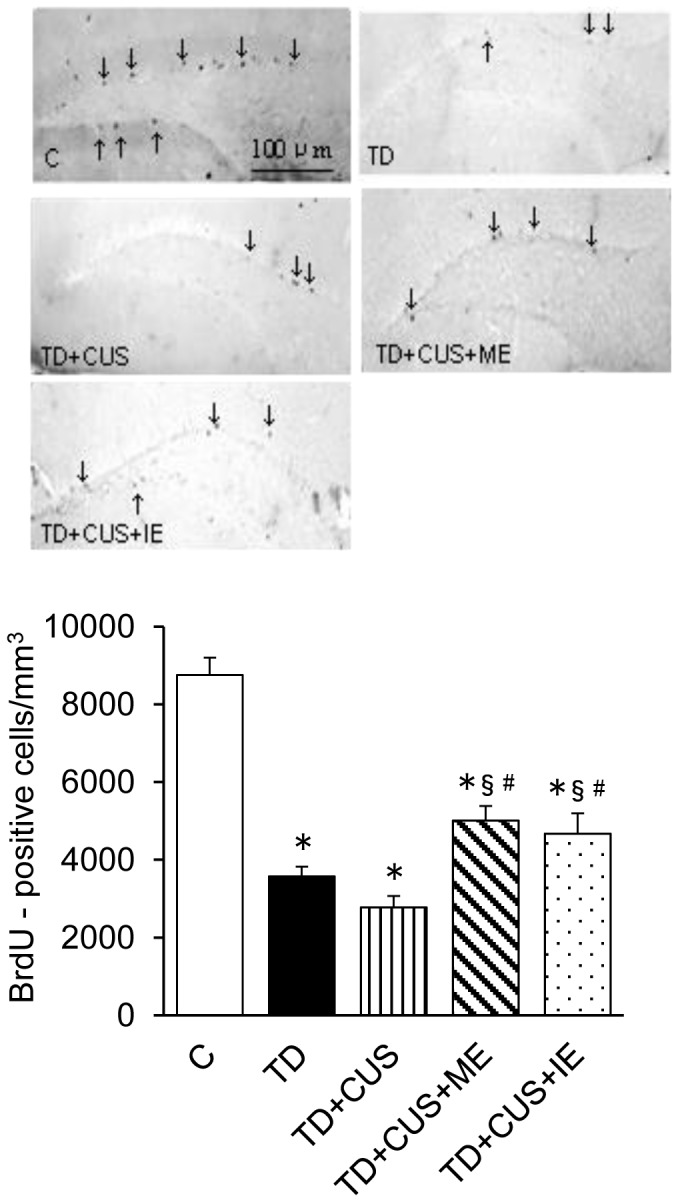
Effects of tryptophan deficiency, CUS and regular exercise on number of BrdU-positive cells in the dentate gyrus of the hippocampus. Data are expressed as mean ± SEM. *, p<0.05 vs. C; §, p<0.05 vs. TD; #, p<0.05 vs. TD+CUS.

The number of Ki67-positive cells, which were used as a marker of proliferating cells [26], [27], was significantly lower in *TD+CUS* mice than in the other mice (F [4], [42] = 4.664; P<0.003) (Fig. 7). No significant difference was discerned, except for *TD+CUS* mice (Fig. 7). On the other hand, the mice fed on TD showed a significantly lower number of BrdU-positive cells than *C* mice (F [4], [39] = 37.78; P<0.001) (Fig. 8). However, comparison among those fed a TD diet revealed that *TD+CUS+ME* and *TD+CUS+IE* mice showed significantly higher numbers of BrdU-positive cells than *TD* and *TD+CUS* (Fig. 8).

## Discussion

To investigate the causal relationship between the level of brain 5-HT and prevention of depression-like behavior via regular exercise, we examined depression-like behavior, brain 5-HT, proliferation and survival of newly born cells in hippocampus, and learning and memory using the mice fed a TD diet and subjected to CUS. The combination of feeding with a TD diet and exposure to CUS led to the development of depression-like behavior, whereas TD alone did not. TD feeding solely impaired learning and memory. Depression-like behavior was prevented by regular exercise, whether moderate or intense, independently of the recovery of brain 5-HT. Regular exercise prevented the impairment of not short-term memory but long-term memory.

Decreases by 40% of hippocampal tryptophan and 5-TH were shown in the mice fed a TD diet (Fig. 3) concomitantly with a 40% decrease in body weight (Fig. 2). These findings suggest that the mice fed a TD diet might supply hippocampal tryptophan by catabolizing skeletal muscle protein into amino acids.

As a decrease of brain 5-HT is considered as the primary cause of the onset and development of major depression, numerous antidepressant drugs that increase the level of brain 5-HT are used for the treatment of depressed patients. These antidepressants prevent the reuptake of 5-HT by the presynaptic neurons and quickly increase the level of brain 5-HT in animals [28], [29], [30]; nevertheless, several weeks of treating patients with these drugs are required for the appearance of a therapeutic benefit [31]. Therefore, the causal relationship between the reduction of brain 5-HT and the onset of depressive behavior is controversial.

Additionally, upon comparison with a previous study, the proportional increase of the immobility time of TD+CUS mice was 37% compared with that of the control mice, whereas in this previous study [16], that of the mice exposed to CUS and fed a normal diet was 34% compared with the control mice. In addition, the survival of newborn cells in the hippocampal dentate gyrus of the TD+CUS mice was 47% lower than that of the control mice, whereas in the previous study [16], that of the mice exposed to CUS and fed a normal diet was 48% lower than that of the control mice. These findings indicate that the reduction of 5-HT leads to no additional impairment in the immobility time of the FST and the survival of newborn cells in hippocampal dentate gyrus. Therefore, we expect that the reduction of 5-HT might not contribute to the onset and development of the depression-like behavior in the CUS model used in the present study.

In this study, however, the combination of feeding on a TD diet and exposure to CUS prolonged the immobility time in FST, whereas feeding on a TD diet alone did not (Fig. 4a). This finding suggested that the onset and development of depression-like behavior in the mice were not attributable to the reduction of brain 5-HT. However, acute tryptophan depletion leads to depression- and anxiety-like behavior in animals [7] and transient mood effects in humans [8]. Why is there a difference in the onset of depression-like behavior between acute and chronic TD? We expect that acute tryptophan depletion leads to a shock state in animals and acute and transient deterioration in cerebral neurotransmitter systems, which cause depression-like behavior or transient mood effects in animals and humans, respectively. In contrast, chronic tryptophan reduction would bring about adaptation to the decreased level of 5-HT and compensation via another neurotransmitter system, which is probably the reason why chronic TD is not linked to the onset of depression-like behavior. However, further study is required to elucidate the etiology of 5-HT in the onset and development of depression.

Previous studies demonstrated that CUS leads to a decrease of sucrose preference in rodents fed a normal diet (Koo et al., Proc Natl Acad Sci U S A 9;107(6):2669-74, 2006). Therefore, it was unexpected that the mice fed a TD diet strongly preferred sucrose solution in spite of CUS exposure (Fig. 4b). This, however, was in agreement with the previous finding that ATD significantly increased the preference for sweet food in humans (Pagoto et al., Eat Behav. 10(1):36–41, 2009). In addition, it has been shown that depressive patients have a strong preference for sweets. At the present time, we do not have an explanation for the fact that mice exposed to a TD diet strongly preferred sucrose solution; therefore, further study is necessary to elucidate the relationship between chronic TD and sweet preference in experimental animals. In contrast, the mice fed on a TD diet strongly preferred sucrose solution in spite of CUS exposure (Fig. 4b), whereas the immobility time of FST was unchanged by no TD feeding but the combination of a TD diet and CUS (Fig. 4a). These findings suggest that SFT may be an inappropriate approach to evaluate depressive behavior in TD animals and that TD alone is not attributable to the onset of depression.

One possible explanation for the exercise-induced prevention of depression-like behavior is the enhancement of hippocampal noradrenaline in the mice that exercised. In this study, regular exercise, whether moderate or intense, prevented the onset of depression-like behavior (Fig. 4). It has been reported that the exercise-induced improvement of major depression is dependent on restoring brain 5-HT [32]. However, in the present study, the hippocampal 5-HT level of the mice that exercised was significantly lower than that of the control mice and equal to that of the stressed mice that did not perform exercise (Fig. 3b). These findings suggested that regular exercise, whether moderate or intense, results in no increase of brain 5-HT, which corresponds with the finding of a previous study that prolonged exercise results in no significant increase of 5-HT level in the brain [33]. Therefore, we think that regular exercise prevents depression-like behavior independent of the level of brain 5-HT. On the other hand, hippocampal noradrenaline was significantly higher in the mice that exercised than in the stressed mice (Fig. 3c). This increase of brain noradrenaline in the exercised mice corresponds to findings in previous studies that the brain noradrenaline level gradually increases with time during prolonged exercise [34], [35]. Brain noradrenaline is a target substance for the pharmacological treatment of depressed patients using serotonin-noradrenaline reuptake inhibitors (SNRIs) and tricyclic antidepressants. Noradrenaline has neuroprotective effects in cultured neuronal cells by stimulating the activation of cAMP-response element binding protein (CREB) and the induction of brain-derived neurotrophic factor (BDNF) [36], [37]. Therefore, we supposed that the enhancement of brain noradrenaline via exercise is a factor that may have contributed to prevent the onset of depression-like behavior in the mice that exercised. In addition, vaccine growth factor (VEG) [38] and vascular endothelial growth factor (VEGF) [16] were identified as other factors related to the prevention of depression-like behavior in animals that exercise. We expect that the antidepressant effect induced by exercise is attributable to complex actions caused by the factors (noradrenaline, VEG, VEGF) influenced by the exercise. Therefore, further investigation is necessary to elucidate the causal relationship between the exercise-induced prevention of depression and these factors.

Another possible factor for examination of the exercise-induced prevention of depression-like behavior is the improvement of the proliferation and survival of newly born cells in the hippocampus of mice that exercised (Fig. 7 and 8). Adult hippocampal neurogenesis is impaired by CUS [39], [40]. A therapeutic effect via antidepressants is concomitant with the improvement of adult hippocampal neurogenesis [41]. Therefore, it is possible that the improvement of adult hippocampal neurogenesis is one of the physiological events that improve depression-like behavior. The present findings demonstrated that regular exercise, whether moderate or intense, restored proliferation (Fig. 7) and the survival of newborn cells in the dentate gyrus of the hippocampus to the normal level (Fig. 8). We suppose that these hippocampal changes might contribute to prevent the onset of depression-like behavior.

The mice fed a TD diet showed impairment of learning and memory without chronic stress (Fig. 5 and 6). These findings suggested that the decrease of brain tryptophan or 5-HT impaired learning and memory, which corresponds to the previous finding that serotonin transporter knockout rats, which showed a lower brain 5-HT level than wild-type rats, exhibited impaired memory as measured by the ORT [42]. These findings indicated that brain 5-HT is an important factor in learning and memory in mice. On the other hand, regular exercise prevented the loss of memory examined by the ORT during the 3rd week of CUS (Fig. 5), which corresponds with the findings of previous studies that regular exercise prevents stress-induced impairment of learning and memory examined by the water maze test [43]; nevertheless, the memory examined by PAT was impaired in the 1st week of CUS (Fig. 6). These findings suggest that regular exercise contributes to prevent not long-term but short-term memory loss. The formation of long-term memory requires the synthesis of several proteins, which include cAMP responsive element binding protein (CBP) [44] and BDNF [45]. As the mice fed on a TD diet could not synthesize these proteins because of *in vivo* TD, they could not avoid the impairment of long-term memory. Further study is required to examine the levels of CBP and BDNF in the brains of mice fed a TD diet.

In summary, the present findings demonstrate that depression-like behavior is attributable not to 5-HT deficiency but to chronic stress. Regular exercise, whether moderate or intense, prevents depression-like behavior with the improvement of hippocampal neurogenesis and without the recovery of hippocampal 5-HT. The impairment of learning and memory is attributable to TD, which is not prevented by regular exercise.

## References

[1] Krishnan V, Nestler EJ (2008) The molecular neurobiology of depression. Nature 455: 894–902. 18923511 10.1038/nature07455 PMC2721780

[2] Jans LA, Riedel WJ, Markus CR, Blokland A (2007) Serotonergic vulnerability and depression: assumptions, experimental evidence and implications. Mol Psychiatry 12: 522–543. 17160067 10.1038/sj.mp.4001920

[3] Cowen PJ, Parry-Billings M, Newsholme EA (1989) Decreased plasma tryptophan levels in major depression. J Affect Disord 16: 27–31. 2521647 10.1016/0165-0327(89)90051-7

[4] Birkmayer W, Riederer P (1975) Biochemical post-mortem findings in depressed patients. J Neural Transm 37: 95–109. 1185163 10.1007/BF01663627

[5] Moja EA, Cipolla P, Castoldi D, Tofanetti O (1989) Dose-response decrease in plasma tryptophan and in brain tryptophan and serotonin after tryptophan-free amino acid mixtures in rats. Life Sci 44: 971–976. 2467158 10.1016/0024-3205(89)90497-9

[6] Stancampiano R, Melis F, Sarais L, Cocco S, Cugusi C, et al (1997) Acute administration of a tryptophan-free amino acid mixture decreases 5-HT release in rat hippocampus in vivo. Am J Physiol 272: R991–994. 9087665 10.1152/ajpregu.1997.272.3.R991

[7] Blokland A, Lieben C, Deutz NE (2002) Anxiogenic and depressive-like effects, but no cognitive deficits, after repeated moderate tryptophan depletion in the rat. J Psychopharmacol 16: 39–49. 11949770 10.1177/026988110201600112

[8] Young SN, Smith SE, Pihl RO, Ervin FR (1985) Tryptophan depletion causes a rapid lowering of mood in normal males. Psychopharmacology (Berl) 87: 173–177. 3931142 10.1007/BF00431803

[9] van Praag HM (2004) Can stress cause depression? Prog Neuropsychopharmacol Biol Psychiatry 28: 891–907. 15363612 10.1016/j.pnpbp.2004.05.031

[10] Gould E, Tanapat P (1999) Stress and hippocampal neurogenesis. Biol Psychiatry 46: 1472–1479. 10599477 10.1016/s0006-3223(99)00247-4

[11] Magarinos AM, Verdugo JM, McEwen BS (1997) Chronic stress alters synaptic terminal structure in hippocampus. Proc Natl Acad Sci U S A 94: 14002–14008. 9391142 10.1073/pnas.94.25.14002 PMC28422

[12] McEwen BS (2004) Protection and damage from acute and chronic stress: allostasis and allostatic overload and relevance to the pathophysiology of psychiatric disorders. Ann N Y Acad Sci 1032: 1–7. 15677391 10.1196/annals.1314.001

[13] Marazziti D, Consoli G, Picchetti M, Carlini M, Faravelli L (2009) Cognitive impairment in major depression. Eur J Pharmacol 626: 83–86. 19835870 10.1016/j.ejphar.2009.08.046

[14] Lavie CJ, Milani RV, O'Keefe JH, Lavie TJ (2011) Impact of exercise training on psychological risk factors. Prog Cardiovasc Dis 53: 464–470. 21545933 10.1016/j.pcad.2011.03.007

[15] Carek PJ, Laibstain SE, Carek SM (2011) Exercise for the treatment of depression and anxiety. Int J Psychiatry Med 41: 15–28. 21495519 10.2190/PM.41.1.c

[16] Kiuchi T, Lee H, Mikami T (2012) Regular exercise cures depression-like behavior via VEGF-Flk-1 signaling in chronically stressed mice. Neuroscience 207: 208–217. 22306286 10.1016/j.neuroscience.2012.01.023

[17] Nakajima S, Ohsawa I, Nagata K, Ohta S, Ohno M, et al (2009) Oral supplementation with melon superoxide dismutase extract promotes antioxidant defences in the brain and prevents stress-induced impairment of spatial memory. Behav Brain Res 200: 15–21. 19373977 10.1016/j.bbr.2008.12.038

[18] Banasr M, Valentine GW, Li XY, Gourley SL, Taylor JR, et al (2007) Chronic unpredictable stress decreases cell proliferation in the cerebral cortex of the adult rat. Biol Psychiatry 62: 496–504. 17585885 10.1016/j.biopsych.2007.02.006

[19] Porsolt RD, Le Pichon M, Jalfre M (1977) Depression: a new animal model sensitive to antidepressant treatments. Nature 266: 730–732. 559941 10.1038/266730a0

[20] Sakata K, Jin L, Jha S (2010) Lack of promoter IV-driven BDNF transcription results in depression-like behavior. Genes Brain Behav 9: 712–721. 20528954 10.1111/j.1601-183X.2010.00605.x

[21] O'Riordan KJ, Huang IC, Pizzi M, Spano P, Boroni F, et al (2006) Regulation of nuclear factor kappaB in the hippocampus by group I metabotropic glutamate receptors. J Neurosci 26: 4870–4879. 16672661 10.1523/JNEUROSCI.4527-05.2006 PMC6674168

[22] De Rosa R, Garcia AA, Braschi C, Capsoni S, Maffei L, et al (2005) Intranasal administration of nerve growth factor (NGF) rescues recognition memory deficits in AD11 anti-NGF transgenic mice. Proc Natl Acad Sci U S A 102: 3811–3816. 15728733 10.1073/pnas.0500195102 PMC553297

[23] Wang H, Ferguson GD, Pineda VV, Cundiff PE, Storm DR (2004) Overexpression of type-1 adenylyl cyclase in mouse forebrain enhances recognition memory and LTP. Nat Neurosci 7: 635–642. 15133516 10.1038/nn1248

[24] Trejo JL, Carro E, Torres-Aleman I (2001) Circulating insulin-like growth factor I mediates exercise-induced increases in the number of new neurons in the adult hippocampus. J Neurosci 21: 1628–1634. 11222653 10.1523/JNEUROSCI.21-05-01628.2001 PMC6762955

[25] Zhang H, Joseph J, Crow J, Kalyanaraman B (2004) Mass spectral evidence for carbonate-anion-radical-induced posttranslational modification of tryptophan to kynurenine in human Cu, Zn superoxide dismutase. Free Radic Biol Med 37: 2018–2026. 15544920 10.1016/j.freeradbiomed.2004.08.026

[26] Kee N, Sivalingam S, Boonstra R, Wojtowicz JM (2002) The utility of Ki-67 and BrdU as proliferative markers of adult neurogenesis. J Neurosci Methods 115: 97–105. 11897369 10.1016/s0165-0270(02)00007-9

[27] Shidara Y, Yamagata K, Kanamori T, Nakano K, Kwong JQ, et al (2005) Positive contribution of pathogenic mutations in the mitochondrial genome to the promotion of cancer by prevention from apoptosis. Cancer Res 65: 1655–1663. 15753359 10.1158/0008-5472.CAN-04-2012

[28] Malberg JE, Eisch AJ, Nestler EJ, Duman RS (2000) Chronic antidepressant treatment increases neurogenesis in adult rat hippocampus. J Neurosci 20: 9104–9110. 11124987 10.1523/JNEUROSCI.20-24-09104.2000 PMC6773038

[29] Marcussen AB, Flagstad P, Kristjansen PE, Johansen FF, Englund U (2008) Increase in neurogenesis and behavioural benefit after chronic fluoxetine treatment in Wistar rats. Acta Neurol Scand 117: 94–100. 18184344 10.1111/j.1600-0404.2007.00910.x

[30] Paizanis E, Renoir T, Lelievre V, Saurini F, Melfort M, et al.. (2009) Behavioural and neuroplastic effects of the new-generation antidepressant agomelatine compared to fluoxetine in glucocorticoid receptor-impaired mice. Int J Neuropsychopharmacol: 1–16. 10.1017/S1461145709990514 19775499

[31] Deltheil T, Guiard BP, Cerdan J, David DJ, Tanaka KF, et al (2008) Behavioral and serotonergic consequences of decreasing or increasing hippocampus brain-derived neurotrophic factor protein levels in mice. Neuropharmacology 55: 1006–1014. 18761360 10.1016/j.neuropharm.2008.08.001

[32] Langfort J, Baranczuk E, Pawlak D, Chalimoniuk M, Lukacova N, et al (2006) The effect of endurance training on regional serotonin metabolism in the brain during early stage of detraining period in the female rat. Cell Mol Neurobiol 26: 1327–1342. 16897368 10.1007/s10571-006-9065-5 PMC11520764

[33] Hasegawa H, Piacentini MF, Sarre S, Michotte Y, Ishiwata T, et al (2008) Influence of brain catecholamines on the development of fatigue in exercising rats in the heat. J Physiol 586: 141–149. 17947314 10.1113/jphysiol.2007.142190 PMC2375558

[34] Hasegawa H, Takatsu S, Ishiwata T, Tanaka H, Sarre S, et al (2011) Continuous monitoring of hypothalamic neurotransmitters and thermoregulatory responses in exercising rats. J Neurosci Methods 202: 119–123. 21683095 10.1016/j.jneumeth.2011.05.024

[35] Takatsu S, Ishiwata T, Meeusen R, Sarre S, Hasegawa H (2010) Serotonin release in the preoptic area and anterior hypothalamus is not involved in thermoregulation during low-intensity exercise in a warm environment. Neurosci Lett 482: 7–11. 20603183 10.1016/j.neulet.2010.06.073

[36] Counts SE, Mufson EJ (2010) Noradrenaline activation of neurotrophic pathways protects against neuronal amyloid toxicity. J Neurochem 113: 649–660. 20132474 10.1111/j.1471-4159.2010.06622.x PMC2913691

[37] Patel NJ, Chen MJ, Russo-Neustadt AA (2010) Norepinephrine and nitric oxide promote cell survival signaling in hippocampal neurons. Eur J Pharmacol 633: 1–9. 20149790 10.1016/j.ejphar.2010.01.012

[38] Hunsberger JG, Newton SS, Bennett AH, Duman CH, Russell DS, et al (2007) Antidepressant actions of the exercise-regulated gene VGF. Nat Med 13: 1476–1482. 18059283 10.1038/nm1669

[39] Li S, Wang C, Wang W, Dong H, Hou P, et al (2008) Chronic mild stress impairs cognition in mice: from brain homeostasis to behavior. Life Sci 82: 934–942. 18402983 10.1016/j.lfs.2008.02.010

[40] Mineur YS, Belzung C, Crusio WE (2007) Functional implications of decreases in neurogenesis following chronic mild stress in mice. Neuroscience 150: 251–259. 17981399 10.1016/j.neuroscience.2007.09.045

[41] Fuss J, Ben Abdallah NM, Vogt MA, Touma C, Pacifici PG, et al.. (2009) Voluntary exercise induces anxiety-like behavior in adult C57BL/6J mice correlating with hippocampal neurogenesis. Hippocampus. 10.1002/hipo.20634 19452518

[42] Olivier JD, Jans LA, Blokland A, Broers NJ, Homberg JR, et al (2009) Serotonin transporter deficiency in rats contributes to impaired object memory. Genes Brain Behav 8: 829–834. 19740092 10.1111/j.1601-183X.2009.00530.x

[43] Nakajima S, Ohsawa I, Ohta S, Ohno M, Mikami T (2010) Regular voluntary exercise cures stress-induced impairment of cognitive function and cell proliferation accompanied by increases in cerebral IGF-1 and GST activity in mice. Behav Brain Res 211: 178–184. 20307585 10.1016/j.bbr.2010.03.028

[44] Chen G, Zou X, Watanabe H, van Deursen JM, Shen J (2010) CREB binding protein is required for both short-term and long-term memory formation. J Neurosci 30: 13066–13077. 20881124 10.1523/JNEUROSCI.2378-10.2010 PMC2953943

[45] Bredy TW, Wu H, Crego C, Zellhoefer J, Sun YE, et al (2007) Histone modifications around individual BDNF gene promoters in prefrontal cortex are associated with extinction of conditioned fear. Learn Mem 14: 268–276. 17522015 10.1101/lm.500907 PMC2216532

